# Magnetic Resonance Imaging in Breast Cancer Tissue In Vitro after PDT Therapy

**DOI:** 10.3390/diagnostics14050563

**Published:** 2024-03-06

**Authors:** Dorota Bartusik-Aebisher, Wiktoria Mytych, Klaudia Dynarowicz, Angelika Myśliwiec, Agnieszka Machorowska-Pieniążek, Grzegorz Cieślar, Aleksandra Kawczyk-Krupka, David Aebisher

**Affiliations:** 1 Department of Biochemistry and General Chemistry, Medical College of the University of Rzeszów, 35-959 Rzeszów, Poland; 2 Students English Division Science Club, Medical College of the University of Rzeszów, 35-959 Rzeszów, Poland; wiktoriamytych@gmail.com; 3 Center for Innovative Research in Medical and Natural Sciences, Medical College of the University of Rzeszów, 35-310 Rzeszów, Poland; kdynarowicz@ur.edu.pl (K.D.); amysliwiec@ur.edu.pl (A.M.); 4 Department of Orthodontics, Faculty of Medical Sciences in Zabrze, Medical University of Silesia, 40-055 Katowice, Poland; apieniazek@sum.edu.pl; 5 Department of Internal Medicine, Angiology and Physical Medicine, Center for Laser Diagnostics and Therapy, Medical University of Silesia in Katowice, Batorego 15 Street, 41-902 Bytom, Poland; cieslar1@tlen.pl; 6 Department of Photomedicine and Physical Chemistry, Medical College of the University of Rzeszów, 35-310 Rzeszów, Poland; daebisher@ur.edu.pl

**Keywords:** MRI, breast cancer, PDT, in vitro

## Abstract

Photodynamic therapy (PDT) is increasingly used in modern medicine. It has found application in the treatment of breast cancer. The most common cancer among women is breast cancer. We collected cancer cells from the breast from the material received after surgery. We focused on tumors that were larger than 10 mm in size. Breast cancer tissues for this quantitative non-contrast magnetic resonance imaging (MRI) study could be seen macroscopically. The current study aimed to present findings on quantitative non-contrast MRI of breast cancer cells post-PDT through the evaluation of relaxation times. The aim of this work was to use and optimize a 1.5 T MRI system. MRI tests were performed using a clinical scanner, namely the OPTIMA MR360 manufactured by General Electric HealthCare. The work included analysis of T1 and T2 relaxation times. This analysis was performed using the MATLAB package (produced by MathWorks). The created application is based on medical MRI images saved in the DICOM3.0 standard. T1 and T2 measurements were subjected to the Shapiro–Wilk test, which showed that both samples belonged to a normal distribution, so a parametric *t*-test for dependent samples was used to test for between-sample variability. The study included 30 sections tested in 2 stages, with consistent technical parameters. For T1 measurements, 12 scans were performed with varying repetition times (TR) and a constant echo time (TE) of 3 ms. For T2 measurements, 12 scans were performed with a fixed repetition time of 10,000 ms and varying echo times. After treating samples with PpIX disodium salt and bubbling with pure oxygen, PDT irradiation was applied. The cell relaxation time after therapy was significantly shorter than the cell relaxation time before PDT. The cells were exposed to PpIX disodium salt as the administered pharmacological substance. The study showed that the therapy significantly affected tumor cells, which was confirmed by a significant reduction in tumor cell relaxation time on the MRI results.

## 1. Introduction 

**Figure 1:**
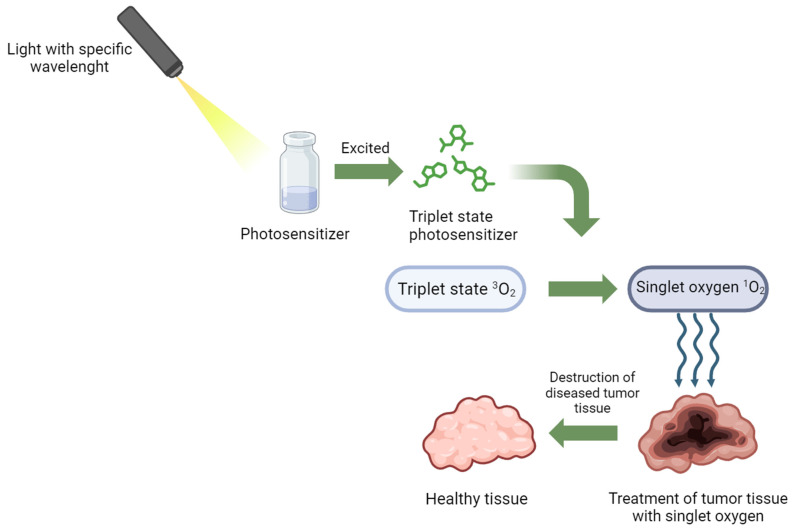
Mechanism of action in PDT therapy.

According to the GLOBOCAN (Global Cancer Observatory) report disclosed by the International Agency for Research on Cancer, in 2020, breast cancer affected 2.26 million individuals, causing 685,000 deaths. Projections for 2040 estimate an increase to 3.19 million cases, including 1.04 million deaths [1]. Although breast cancer is the most common cancer among women worldwide, it is infrequent in men and constitutes only 1% of male cancer cases with a mortality rate below 0.2%. Breast cancer research predominantly focuses on female cases. Timely diagnosis significantly influences treatment choices, effectiveness, and post-treatment prognosis [2,3,4]. Unfortunately, many breast cancer diagnoses occur at advanced stages, impacting the likelihood of favorable outcomes. However, a 2003 study by Ellis et al. found positive prognoses in some histologically advanced breast cancers. Screening mammography is commonly used for diagnosis, followed by mastectomy or breast-conserving surgery, complemented by radiation therapy, chemotherapy, and hormonal therapies [5,6,7]. Despite these approaches, there is a continuous search for new technologies, such as photodynamic therapy (PDT), as an alternative treatment for non-responsive or ineligible patients [2,6,8,9]. PDT involves three stages: step (1) is the introduction and accumulation of photosensitizer at the target site; step (2) is light irradiation to excite the photosensitizer; step (3) is the presence of dissolved oxygen in the tissue (Figure 1). The effectiveness of PDT depends on certain factors, like the photosensitizer’s location and type, dosage, light exposure, oxygen levels, and the time between photosensitizer administration and illumination. PDT’s therapeutic agent responds only when it is localized in the treated tissue areas [10,11,12,13].

In the classification of breast cancer, a very important aspect is the degree of tumor differentiation, pleomorphism, and proliferation, which form the basis on which the histological grade of the lesion is determined [14,15,16]. The WHO divides breast cancer into 20 main types and 18 subtypes. Invasive breast cancers are divided into specific and non-specific types; non-specific breast cancers account for 60–75% of all cancers and specific types account for 20–25% [17,18,19]. Traditionally, breast cancers were classified by morphology. Today, scientists are studying the division using molecular classification, which has become a developing branch of medicine [20,21,22,23]. Scientists agree that breast cancer is a heterogeneous disease entity that varies phenotypically. Thus, a molecular classification has been developed based on the traditional morphological classification [24,25,26,27,28] (Table 1).

### 1.1. Risk Factors 

After the age of 40, regular mammograms are recommended for women. This is related to the increasing risk of breast cancer incidence with age. Scientific studies show a high incidence rate in women over 40 [37,38]. 

The second most important factor associated with the incidence of breast cancer is the gene gent mutation of BRCA1 and BRCA2. Studies estimate that 25% of cases are associated with a family history. The risk of incidence increases significantly in women with a familial background of breast cancer [39,40,41,42]. 

Further elements of risk factors are reproductive factors. These include menopausal age, age of pregnancy, and number of pregnancies. Studies estimate that each pregnancy reduces the risk of breast cancer by about 5–10%. And, each year of delayed menopause increases the risk by 3%. Norwegian studies show that pregnancy under 20 and over 35 can increase the risk of breast cancer [43,44,45].

Estrogen is also linked to breast cancer risk. Endogenous estrogen is produced by the ovaries, and exogenous estrogen usually comes from contraceptives and hormone replacement therapy [46,47,48].

Another crucial factor is the contemporary way of life. Research indicates that smoking, particularly during youth, is linked to a higher occurrence of breast cancer. Additionally, a diet rich in fats, especially saturated fats, and excessive alcohol consumption play significant roles in breast cancer occurrence. Studies demonstrate that ingesting 35–44 g of alcohol daily raises the likelihood of developing breast cancer by 32% [49,50,51].

### 1.2. Nanomaterials in Breast Cancer Therapy 

Nanomaterial-based therapeutic approaches for breast cancer represent a rapidly evolving field that leverages the unique properties of nanoscale materials for diagnosis, imaging, and treatment. These approaches aim to enhance the specificity and effectiveness of breast cancer treatments while minimizing side effects. Various types of nanoparticles, such as liposomes, polymeric nanoparticles, and dendrimers, can be designed to encapsulate chemotherapeutic agents. These nanocarriers protect the drug during circulation, improve its pharmacokinetics, and allow for targeted delivery to cancer cells, minimizing damage to healthy tissues [52]. Amphiphilic block copolymers can self-assemble into micelles, which can encapsulate hydrophobic drugs. Micelles offer improved drug solubility and stability, aiding in controlled drug release [53,54]. Iron oxide nanoparticles can be guided to tumor sites using an external magnetic field. Upon gathering within the tumor, these nanoparticles can produce heat when subjected to an alternating magnetic field, causing hyperthermia and selectively harming cancer cells [55]. Gold nanoparticles can absorb near-infrared light (NIR) and convert it into heat, selectively destroying cancer cells through hyperthermia. This photothermal effect is harnessed for targeted tumor ablation [56]. Nanoparticles can function as contrast agents in diverse imaging methods, such as magnetic resonance imaging (MRI), computed tomography (CT), and photoacoustic imaging. These agents improve the visualization of tumors and aid in early diagnosis [57,58]. Semiconductor nanocrystals known as quantum dots emit specific wavelengths of light based on their size. They have been explored for fluorescence imaging, providing a sensitive and versatile tool for cancer detection [59]. Lipid-based nanoparticles can be utilized for the delivery of therapeutic nucleic acids, such as small interfering RNA (siRNA) or microRNA. These nanoparticles protect the nucleic acids from degradation and facilitate their intracellular delivery for gene silencing or modulation [60]. Nanoparticles can be engineered to carry tumor-associated antigens or immunomodulatory agents, enhancing the effectiveness of cancer vaccines and stimulating the immune system to recognize and attack cancer cells [61]. Nanomaterial-based approaches hold great promise for advancing the precision and efficacy of breast cancer therapies. However, it is crucial to note that the field is still evolving, and extensive research, including preclinical and clinical studies, is ongoing to validate the safety and effectiveness of these nanomaterial-based therapeutic strategies. Nanomaterials for artificial tissue contexts have become a promising avenue for breast cancer treatment. As nanotechnology advances, understanding the effects of specific dopants on the thermal response of artificial tissues is becoming crucial to designing effective and targeted therapies. Valuable information on this topic is presented by A. Gaona-Esquivel et al., and this study addresses the key role of Nd as a dopant in Mn_3_O_4_NPs (manganese oxide nanoparticles) and its effect on heat induction in artificial breast tissue when subjected to microwave radiation. By delving into the intricacies of this study, we gain insight into the nuanced interactions between nanomaterials and target tissues, particularly in the context of breast cancer therapy [62].

### 1.3. Protoporphyrin IX Disodium Salt

Protoporphyrin IX (PpIX) disodium salt is a chemical compound that is related to the heme group, which is an essential component of hemoglobin and other hemoproteins. Heme is an intricate organic compound that incorporates iron, and it serves a vital function in numerous biological mechanisms, such as oxygen transportation and electron transfer. PpIX disodium salt is a key component in the field of PDT [63]; it serves as a photosensitizer (PS) and is often used because it has a high affinity for cancer cells. This PS has a propensity to accumulate within targeted cancer cells. Following the administration of PS, the affected area is then exposed to light, typically of a specific wavelength that corresponds to the absorption peak of PpIX disodium salt. Upon light exposure, PpIX disodium salt undergoes a photochemical reaction, resulting in the creation of reactive oxygen species (ROS). The production of ROS within the targeted cells results in a cascade of cellular events, ultimately resulting in harm to cellular structures and culminating in apoptosis or necrosis of the treated cells. Importantly, this process is selective, as PpIX disodium salt tends to accumulate more in abnormal or cancerous cells compared to healthy tissue [64,65]. Ongoing research continues to explore and refine the application of PpIX disodium salt in PDT, expanding its potential therapeutic uses and improving its clinical outcomes.

### 1.4. Light-Based Breast Cancer Techniques

Light-based techniques are being explored for breast cancer detection and treatment. Diffuse optical imaging is a non-invasive imaging method that utilizes NIR light to generate three-dimensional (3D) images of breast tissue [66]. It helps in detecting changes in tissue properties associated with breast cancer [67]. Photoacoustic imaging combines laser-induced ultrasound and optical imaging. Laser pulses generate ultrasound waves when absorbed by tissues, and the resulting acoustic signals are used to create images. Photoacoustic imaging can provide functional and molecular information about breast tissue [68]. Fluorescence-based imaging involves the use of fluorescent dyes that selectively bind to cancer cells. When exposed to light of a specific wavelength, these dyes emit fluorescent signals, helping to identify and locate cancerous tissues [69]. Elastography is not purely light-based. Elastography often involves the use of ultrasound and optical techniques to assess tissue stiffness. Changes in tissue stiffness can be indicative of the presence of tumors, including breast cancer [70]. Raman spectroscopy uses laser light to analyze the molecular composition of tissues. It can provide detailed information about the biochemical and molecular changes associated with cancer [71]. Kothari R. et al. explored notable progress in the application of Raman spectroscopy for diagnosing breast cancer surgically in their study. By measuring the inelastic scattering of photons, this method provides valuable information about molecular structures and chemical tissue compositions. The authors show that many researchers can achieve high predictive accuracy in distinguishing between normal and cancerous breast tissue. However, sensitivity and specificity tend to decrease in studies with larger sample sizes. Contributing factors include differences in menstrual status affecting the optical characteristics of breast tissue, the ratio of lipid tissue to fibrous tissue found in normal, healthy breasts, the density of tumor cells in the healthy matrix, and the presence of surgical ink after lumpectomy procedures [72]. Optical coherence tomography uses low-coherence light to create high-resolution, cross-sectional images of tissues. It has been explored for its potential in detecting early-stage breast cancer by visualizing the tissue microstructure. In light-based techniques for breast cancer, different light sources are used depending on the specific imaging or treatment modality. Some common light sources employed in the context of breast cancer diagnosis and treatment are as follows. In NIR imaging, light is often used in diffuse optical imaging and spectroscopy techniques. NIR light penetrates tissues more effectively than visible light, allowing for non-invasive imaging and analysis of tissue properties. Diode lasers emitting in the NIR range are commonly used in these applications [73]. Laser diodes are compact and efficient sources of coherent light. They are used in various optical techniques, such as PDT and fluorescence imaging. Laser diodes can emit light at specific wavelengths, enabling targeted interactions with tissues or contrast agents [74]. Pulsed lasers are used in photoacoustic imaging. These lasers emit short pulses of light, typically in the nanosecond or picosecond range, inducing acoustic waves in tissues. The generated acoustic signals provide details regarding tissue composition and can aid in pinpointing areas of significance [75]. Light-emitting diodes are employed in some optical imaging techniques due to their cost-effectiveness and ease of use. While not as intense as lasers, LEDs can still provide sufficient light for certain imaging applications, especially when using non-coherent light [76]. In fluorescence imaging, diverse light sources, such as mercury vapor lamps or light-emitting diodes, are employed to activate fluorophores. The emitted fluorescence is then detected and used to visualize specific molecular targets associated with breast cancer [77]. The choice of light source depends on the specific requirements of the imaging or treatment method, including the desired penetration depth, tissue interactions, and the spectral properties needed for detection. Researchers and clinicians carefully select the appropriate light source based on the goals of the procedure and the characteristics of the tissues being studied or treated. Taha S. et al. explored the effects of femtosecond laser exposure on breast cancer, utilizing the T47D cell line as an in vitro representation. Femtosecond laser exposure at specific wavelengths, especially 380 and 400 nm, demonstrated significant inhibition of breast cancer cell growth. The effect was observed both immediately and 24 h after exposure, with increased efficacy noted at longer exposure times, particularly 10 min. Cell viability was not significantly influenced by wavelengths of 700, 720, 750, and 780 nm. Overall, femtosecond laser irradiation exhibited a noteworthy anticancer effect against T47D cells, suggesting its potential use in breast cancer management [78]. 

### 1.5. Benefits and Drawbacks

Each of these approaches has its benefits and drawbacks, and the selection of treatment is contingent upon such factors as the kind and stage of breast cancer, as well as the overall health of the patient and individual preferences (Table 2). It is important to note that the benefits and drawbacks mentioned are generalizations, and individual experiences can vary. Treatment decisions should be made in consultation with a multidisciplinary healthcare team, considering the unique characteristics of the patient and the specific features of the cancer. Additionally, ongoing research and advancements may lead to new treatment options with improved efficacy and reduced side effects.

### 1.6. PDT Therapy in Breast Cancer 

The concept of PDT dates to the early 20th century, but serious investigations into its application for cancer treatment began in the 1970s and 1980s. Researchers began studying the use of photosensitizing agents and light for the selective destruction of cancer cells. In the 1990s, notable advancements were made in creating specialized photosensitizing agents intended for photodynamic therapy (PDT). Numerous studies were conducted to assess the efficacy of different photosensitizing agents in the context of PDT for breast cancer [93]. Porphyrins, chlorins, and phthalocyanines were among the first-generation photosensitizers explored for breast cancer PDT [94]. Advances in laser technology, imaging, and our understanding of cancer biology contributed to the refinement of PDT. DICOM (digital imaging and communications in medicine) is primarily associated with medical imaging data, including radiology, cardiology, and other diagnostic modalities. DICOM standardizes the communication and management of medical imaging information and images, ensuring interoperability and consistency in healthcare systems. While DICOM is not traditionally linked to PDT, the integration of imaging data and standardization can have implications for treatment planning, monitoring, and research in PDT. DICOM images obtained from various imaging modalities, such as MRI, CT, or optical imaging, can be used for treatment planning in PDT [95]. Integration of DICOM images may help in precisely targeting the photosensitizing agent to the tumor site during PDT. DICOM images acquired before, during, and after PDT sessions can provide a standardized means of monitoring treatment response. Changes in tumor size, vascularity, or other relevant parameters can be documented and analyzed using DICOM. DICOM data from imaging studies can be crucial in the design and analysis of clinical trials involving PDT. Standardized image data allow for consistent evaluation of treatment outcomes and comparison across different studies. PDT may be part of a multimodal treatment approach, combining various imaging techniques. DICOM facilitates the integration of data from different modalities. Multimodal imaging data can enhance the understanding of the treatment response and guide subsequent therapeutic decisions. DICOM standardization ensures the quality and consistency of imaging data, supporting quality assurance in PDT procedures. Documentation of treatment sessions using DICOM metadata can be valuable for auditing, quality improvement, and retrospective analysis. It is essential to note that the direct incorporation of DICOM in PDT may depend on the specific imaging techniques used in PDT research and clinical practice. Researchers and clinicians may leverage DICOM standards when using imaging modalities, such as CT, MRI, or optical imaging in the context of PDT [96,97,98]. Bayareh-Mancilla R. et al. developed a computational framework for detecting asymmetry in mammographic images using dynamic time warping (DTW) for shape analysis and the growing seed region (GSR) method for breast skin segmentation. The study achieved an 83% accuracy in identifying potential asymmetry cases in skin thickness. The growing seed region (GSR) method was validated with high similarities (90.47% and 66.66%) for asymmetry cases compared to ground truth segmented images. The system successfully identified 35 patients with potential skin asymmetry. A graphical user interface (GUI) was designed to enhance accessibility for physicians, allowing the input of DICOM files and providing visual representations of asymmetrical findings. In summary, the computational system combines DTW and GSR methods, demonstrating promising accuracy and offering a user-friendly interface for effective validation and interpretation of mammographic images by physicians. In summary, the research introduces a computer system combining DTW and GSR methods for asymmetry detection, achieving promising accuracy and offering a user-friendly interface for physicians to validate and interpret mammographic images effectively [99]. 

Researchers gained insights into the mechanisms underlying PDT and its effects on cancer cells. In vivo studies conducted in living organisms, including animal models and eventually human trials, provide crucial insights into the efficacy and safety of PDT. Animal studies involving breast cancer xenografts or spontaneous tumors have shown promising results, with PDT leading to tumor regression and improved survival rates. 

Liu Y. et al. assessed the synergistic impacts of sonodynamic therapy (SDT) and PDT using DVDMS in vitro and in vivo on breast cancer. In the in vitro experiments, DVDMS-SPDT (sinoporphyrin sodium sono-photodynamic therapy) demonstrated significantly more pronounced cytotoxicity when compared to the individual applications of SDT or PDT, as determined through MTT and colony formation assays. Analysis using 2′,7′-Dichlorodihydrofluo-rescein-diacetate and dihydroethidium staining indicated a substantial increase in intracellular ROS in groups subjected to the combined therapy. Examinations using the terephthalic acid method and FD500 uptake assay indicated that the increased efficacy of the combined therapy was also influenced by the effects of cavitation and changes in cell membrane permeability resulting from ultrasound irradiation. The findings from the study propose that the integration of SDT and PDT, particularly with the DVDMS sensitizer, may result in significantly enhanced therapeutic outcomes, showcasing DVDMS-SPDT as a potential strategy against highly metastatic breast cancer [100].

The same conclusion was reached by Wang X. et al. in their in vivo and in vitro research arrived at a similar outcome when examining the photodynamic impact of DVDMS on 4T1 breast cancer. The findings indicate that DVDMS exhibited greater efficacy in suppressing cancer proliferation and spreading, in contrast to PF, a traditional photosensitizer. widely used in clinical settings [101]. Articles confirm the significant effect of the DVDMS method. The following study introduces a hopeful strategy for addressing triple-negative breast cancer (TNBC) through the utilization of a cancer cell membrane-coated oxygen delivery nanoprobe known as a cancer cell membrane-coated indocyanine green-doped perfluorocarbon (CCm-HSA-ICG-PFTBA).

This nanoprobe effectively targets TNBC tissues, alleviates tumor hypoxia, and enhances the efficacy of PDT in TNBC xenografts. The use of FDA-approved materials, including human serum albumin (HSA), indocyanine green (ICG), and perfluorocarbon (PFC), ensures high biocompatibility and potential for clinical translation. The in vitro and in vivo results demonstrate improved oxygen delivery, increased ROS concentrations, and a significant reduction in tumor volume and weight, highlighting the nanoprobe’s therapeutic potential for TNBC treatment. Additionally, the absence of observed biotoxicity up to 14 days post-injection is a positive safety indicator for further development and clinical applications [102]. Liu XL. et al. investigated the tumorigenic potency of liposomes in an orthotopic 4T1 mouse breast tumor model. The photosensitizer verteporfin was loaded in the lipid bilayer to confer PDT activity. The study showed about 90% inhibition of tumor growth, and survival was prolonged by 72% [103].

**Figure 2:**
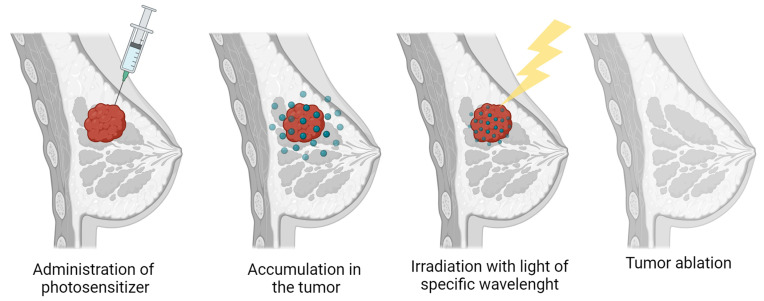
PDT therapy in breast cancer.

In vitro studies involving breast cancer cells have demonstrated the potential of PDT to induce cell death selectively in cancerous cells. These studies often involve exposing cultured cancer cells to photosensitizing agents and light in controlled laboratory settings. In vitro experiments help researchers understand the mechanisms of PDT and optimize treatment parameters. Chota A et al. in their study showed that the effects of plant D.anomala root extract and ZnPcS4 mediated PDT in Michigan Cancer Foundation-7 (MCF-7). The results indicate enhanced cytotoxicity and the possibility of a reduced dose of photosensitizer with preserved effects [104]. To evaluate the cytotoxicity and affinity of breast and prostate cancer cells for application in PDT, hyperthermia therapy, and PET/MR imaging, the following study synthesized Fe_3_O_4_@TiO_2_ nanoparticles tagged with 89Zr. The results presented here shed further light on the interactions between 89Zr-Df-Bz-NCS that are linked via nanoconjugates. They also offer intricate speciation and stability data, which could be useful in the creation of more effective PET chelators. The authors demonstrate how these nanoparticles may improve the sensitivity and accuracy of lesion identification [105]. The second case involved using the MCF-7/MDR line for chemo-resistant breast cancer treatment. They employed factor VII PDT (fVII-tPDT) with Sn (IV) chlorin e6, targeting both tumor cells and vascular endothelial cells. fVII-tPDT exhibited 12 times higher efficacy in vitro compared to ntPDT, leading to cell death and necrosis. FVII-tPDT has been shown to be a safe and effective treatment for chemo-resistant breast tumors in a model of naked mice. The researchers concluded that fVII-tPDT is safe and effective in treating chemo-resistant breast cancer because it presumably targets tumor neovascularization and chemo-resistant tumor cells simultaneously [106]. Zhu J. et al. demonstrated the efficacy of MPPa-PDT in inhibiting the growth of human MDA-MB-231 breast cancer cells in vitro. The study highlights the positive impact of PDT therapy, as confirmed by the analysis of the apoptotic index [107]. Peng Z. et al. examined the in vitro effects of PDT on human breast cancer cells MCF7 and MDA-MB-231, which were mediated via a hematoporphyrin derivative (HPD). To increase PpIX fluorescence and the HPD-PDT response, this study [108] demonstrated a simple, safe, and highly successful preconditioning method for breast cancer patients. Applying AuNPs to hydrophobic PS, such as hypericin, improves the efficacy of PDT and mostly induces cell death [109]. It has been demonstrated that the Ce6-PC-Tmab@A-Gel System, with its favorable NIR response release characteristics, is an effective targeted therapy. The results of this study suggest that a medication may be released locally in the tumor for a prolonged duration, potentially improving the efficacy of treatment for drug-resistant breast cancer [110]. Clinical trials exploring the use of PDT in breast cancer patients began to emerge in the 2000s. These trials aimed to assess the safety, efficacy, and optimal parameters for PDT in human subjects. Ongoing research continues to explore the potential of PDT in breast cancer treatment. Clinical trials are investigating PDT in combination with other therapies or as part of a multimodal treatment approach. While PDT for breast cancer has shown promise in preclinical studies and early-phase clinical trials, it has not yet become a standard treatment. The field is dynamic, with ongoing efforts to optimize PDT protocols, enhance specificity, and improve overall therapeutic outcomes. In summary, the history of PDT in breast cancer reflects a progression from early explorations to ongoing research and clinical trials. As the field advances, PDT might assume a growing significance in the comprehensive care of breast cancer. Figure 2 shows individual stages of breast cancer treatment using PDT therapy. 

### 1.7. MRI in Breast Cancer In Vitro 

Utilizing an MnAs-ICG nanospike in their investigation, Chen X. et al. demonstrated their double pH/photothermal discharge instrument. The MRI comes from in vitro and in vivo tests, and they recommend the potential of MnAs-ICG as a promising tool that can specifically differentiate cells for the exact discovery and treatment of breast cancer through MRI [111]. 

In vitro, Chauhan R. et al. demonstrated the successful functionalization of gold nanoparticles (GNP-Gd(III)-DO3A-SH-) with the targeted oligonucleotide AS1411 in breast cancer cells. This led to a significant increase in the contrast agent uptake for MRI imaging in aggressive MDA-MD-231 cells compared to untargeted control counterparts [112]. 

In a study using quantitative cellular MRI, Alhamami M. et al. demonstrated that MnPs (manganese porphyrins) have the potential for sensitive imaging across various clinical breast cancer cell subtypes. This is attributed to their enhanced relaxivity, cellular uptake, and retention when compared to Gd-DTPA. The research also emphasized the promising capabilities of a more hydrophobic MnP, MnTPPS3NH2, as a T1 contrast agent for the sensitive detection of various breast cancer cells in vitro using quantitative cellular MRI [113]. 

Moradi Khaniabadi P. et al. connected C595 monoclonal antibodies conjugated to superparamagnetic press oxide nanoparticles (SPIONs-C595) for the location of breast cancer cells (MCF-7). In vitro MRI illustrated a 76% lessening in T2 unwinding time utilizing T2-weighted MRI pictures compared to the control bunch at the ideal dosage of 200 μg Fe/mL. The comes about demonstrated a tall nanoprobe take-up into MCF-7 tumor cells, emphasizing the SPIONs-C595 nanoprobe’s potential for the tall T2-weighted MRI to differentiate early-stage breast cancer in vitro [114]. 

Conducting in vitro and in vivo examinations, Savolainen H. et al. confirmed the capability of using PET-MRI in conjunction with 68Ga-Sienna+ to localize and characterize sentinel lymph hubs by employing a mouse model of breast cancer with unconstrained lymph hub metastases. The considered study illustrated that 68Ga-Sienna+ PET-MRI can serve as a profitable preoperative imaging instrument [115]. 

Sharma U. and Jagannathan NR. detailed discoveries recommending that including MRS examination improves the specificity of MRI. In vitro and ex vivo 1H MRS offer a comprehensive biochemical characterization of intaglio breast cancer tissues or extricates [116]. 

Alberti D. et al. utilized MRI to illustrate that a carbonic anhydrase inhibitor containing carborane can hinder the development of mesothelioma and breast cancer cells, based on its official response to CAIX overexpressed in these cells [117]. 

Jansen SA. et al. determined that tiny, early mammary cancers in mice, counting DCIS, can be precisely identified by MRI. In an in vitro study, mice experienced imaging utilizing T2-weighted turn reverberate groupings. The creators showcased that MR imaging dependably recognizes both pre-invasive in situ and early obtrusive breast cancers in mice with tall affectability [118].

In their study, Li XB. et al. used a magnetic resonance (MR) molecular probe for the somatostatin receptor expressed on breast cancer cell membranes and investigated its physicochemical properties and imaging characteristics in vitro. They showed that the molecular probe could effectively label breast cancer cells expressing SSTR [119].

## 2. Materials and Methods 

**Figure 3:**
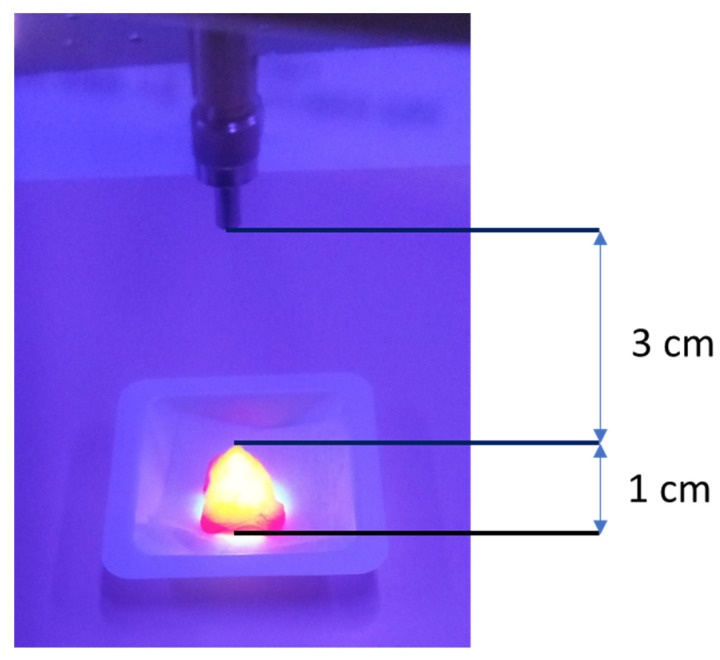
Irradiation set up.

The aim of the study was application of an MRI method to evaluate in vitro breast cancer tissue sections after PDT therapy with PpIX disodium salt, based on changes in T_1_ and T_2_ relaxation time values. All investigations were carried out with approval (No. 10/11/2018) from the Bioethics Committee at the Rzeszów University. Patients included within the study were analyzed and clinically organized as IIB or III (locally progressed and metastatic breast cancer, respectively) through center needle biopsy and vacuum-assisted biopsy. The spin–lattice (T1) and spin–spin (T2) relaxation times were surveyed by employing a Tesla Optima MR360 attractive reverberation imaging gadget from Common Electric Healthcare (Milwaukee, WI, USA). The device worked with SV23 computer program elucidation. The chosen tests was experienced by utilizing quick spin echo (FSE) arrangements with hub projection utilizing a little flex coil. DICOM pictures were analyzed, and ROI measures were taken for an arrangement of pictures for each test. Hence, T1 and T2 unwinding times were decided based on the obtained information. In total, 30 segments were inspected in 2 diverse stages of the study. The specialized parameters for the MRI studies utilizing the attractive reverberation gadget remained steady through all stages of the study, with a filtering network of 320 × 224, segment thickness of 2 mm, remove of 0.5 mm, and NEX = 2. For T1, measures were conducted in 12 steps with a redundancy time (TR) extending from 50 to 15,000 ms, and a reverberate time (TE) of 3 ms. For T2, an arrangement of 12 steps was performed with a settled reiteration time of 10,000 ms and a reverberate time extending from 8.7 to 250 ms. Each test with PpIX disodium tar was exposed to immaculate oxygen for 2 min, and 1 mL was added into the segments for photodynamic treatment. The tests were lighted with a 665 nm wavelength bar at a power of 350 mW from a distance of 10 cm to encourage warming. The control was measured by employing a control meter. To initiate photodynamic activity, a solid-state laser (LD Pumped All-Solid-State Green Laser, MGL-III-532 nm/300 mW) coupled to a fiber optic cable was utilized to convey 532 nm light to the treated tissue tests for 15 min (Figure 3).

The duration from after the PDT slides were prepared and vanquished to the time of glamorous resonance imaging was used to determine T1 and T2 relaxation times. The scanning parameters used in this stage of the study were unchanged from the first round.

## 3. Results

Statistica software (version 13.3, Statsoft, Kraków, Poland) was used for statistical analysis. Graphical processing of the results was prepared using Microsoft Excel (version 16.0) and Microsoft Word (version 16.0). In the analysis, descriptive statistics, including count, mean, standard deviation, median, minimum, and maximum values, and upper and lower quartiles, were employed. The normality of the data distribution was evaluated using the Shapiro–Wilk test. Based on the results of these tests, appropriate tests were selected to analyze the differences between the study groups. A significance level of alpha = 0.05 was adopted; this implies that outcomes were deemed statistically significant if the *p*-value was below 0.05. The study investigated T1 and T2 relaxation times in breast tissue before and after PDT using spin—lattice mapping. T1 time reflects changes in cellular and extracellular components, influenced by various factors, while T2 time helps to distinguish healthy from diseased tissue based on the decay of transverse magnetization.

Using MRI, 30 samples containing PDT-treated breast cancer tumor cells were examined. Each of the studied samples contained 30 observations each; the measurements of sample one were in the range of 505–598 ms, and the mean was also very close to the median. Sample two was in the range of values between 34 and 117 ms. The ranges of measurements, therefore, differed very strongly between the two samples. Also, the uncertainties in the case of the averages differed by more than a factor of two, while in the case of the median the difference was four times (Table 3). 

**Figure 4:**
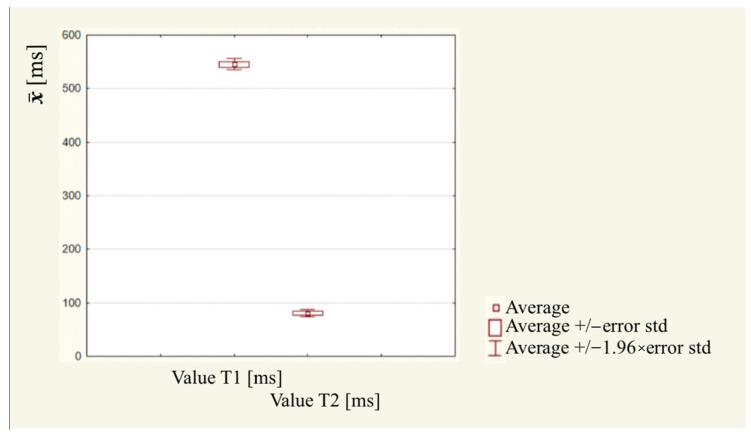
Box and whisker plot measurement of T1 and T2 values between the pre- and post-PDT therapy measurements.

T1 and T2 measurements were subjected to the Shapiro–Wilk test, which shows that both samples belong to a normal distribution, so a parametric *t*-test for dependent samples was used to test the variation between samples. Using a statistical significance result (*p* < 0.001), statistically significant differences were shown between the pre- and post-therapy measurements. The relaxation time of cells after therapy was significantly lower than that of cells before PDT therapy. The difference in means is 464.5 ms, while the standard deviation differs by nearly 10 ms (Table 4). The measurements of the second sample, therefore, are, on average, more than six times lower and are much more concentrated around the mean, as can be observed in the box and whisker plot (Figure 4). 

**Figure 5:**
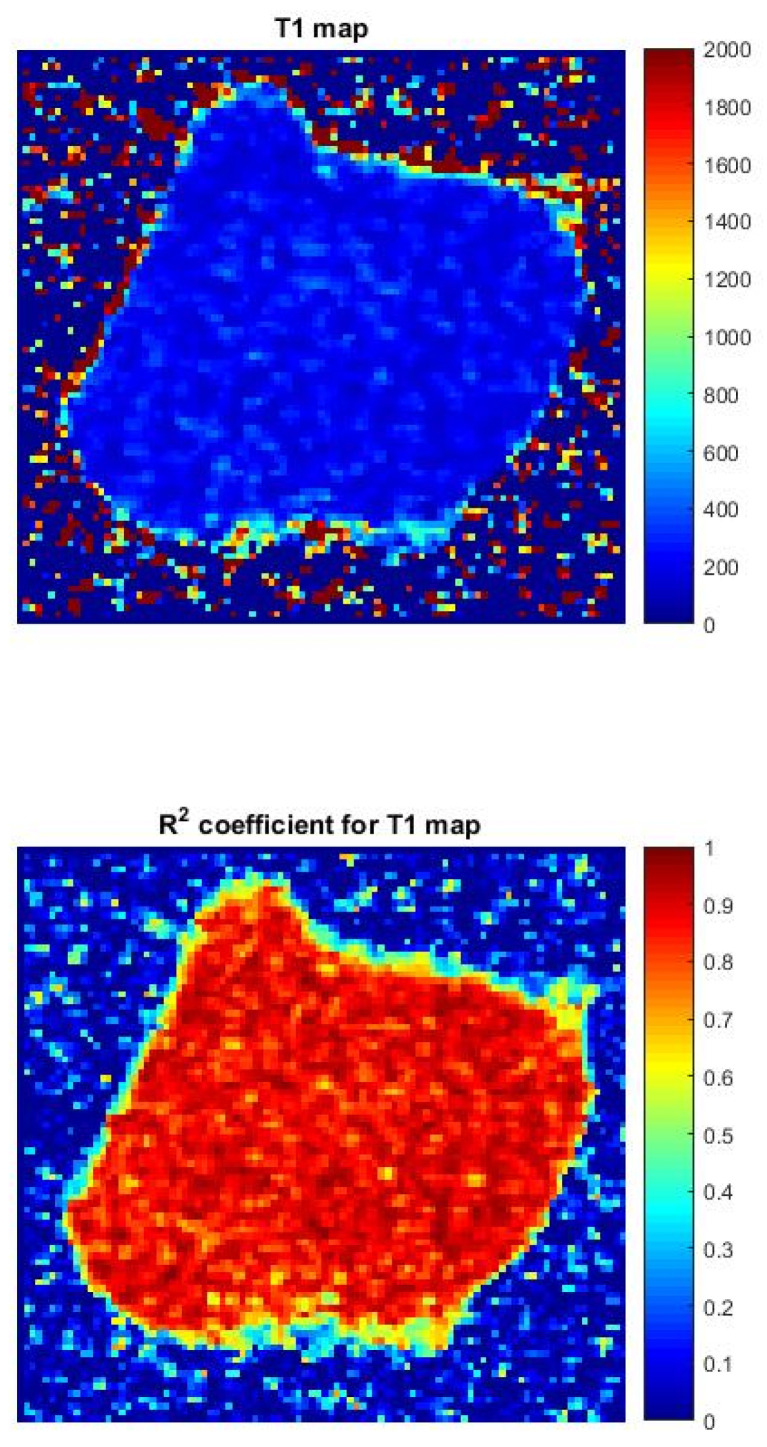
An example of a T1 map of breast cancer and the R2 coefficient for a T1 map.

The results obtained showed a statistically significant decrease in cell relaxation time after PDT treatment compared to the pre-treatment state, with a mean T1 relaxation time of 545.27 ms and a mean T1 relaxation time of 545.27 ms. T2 relaxation is 80.77 ms. Application of the Shapiro–Wilk test confirmed the normal distribution of the relaxation time data. A dependent samples *t*-test further highlights the significance of the observed difference (*p* < 0.001). This reduction in relaxation time, indicating altered tissue properties, has a significant impact on tumor cells, consistent with the general conclusion that PDT with the disodium salt of PpIX has a marked effect on causing cell death in breast cancer. Consistent results from different studies support the potential of PDT as a minimally invasive, targeted treatment option for breast cancer, demonstrating its ability to impact relaxation time, and it serves as a measure of treatment effectiveness. Figure 5 shows an example of a T1 map of breast cancer and the R2 coefficient for a T1 map.

## 4. Discussion

Annually, over 1.5 million women worldwide receive a diagnosis of breast cancer. The prognosis for breast cancer is predominantly influenced by the timing of disease detection and the clinical subtype of the cancer [120,121]. Diagnosis has a central role in breast cancer prevention, and early detection of cancer is the foundation of prevention. A diagnostic option that is non-invasive and widely available to every woman is breast self-examination, which every woman should perform once a month. Basic diagnostic tests include breast ultrasound and mammograms. Studies suggest a connection between undergoing screening mammography and a reduction in breast cancer mortality rates among women within the age group of 40 to 49 years [122]. Patients with breast cancer or an inconclusive result or suspected precancerous lesions may be offered tests, such as contrast-enhanced MRI, coarse-needle biopsy, galactography, pneumocystography, or fine-needle aspiration in addition to clinical examination, mammography, and ultrasound. The advantage of MRI is that the test result is not affected by breast density and the test itself allows for detection of lymph node metastases [123]. Genetic testing could be offered to women with a familial history of breast cancer due to the identification of the BRCA1 and BRCA2 genes, mutations which may be associated with the development of breast cancer [124]. Among treatment methods, one of the most common is surgical treatment. This treatment can be divided into breast-sparing methods, which include lumpectomies and quadrantectomies, and breast amputations, namely mastectomies. Radiation therapy is another of the breast cancer treatments, which is mainly used as an adjunct to breast-sparing treatment. Complementary treatment for breast cancer is used depending on the size of the tumor, lymph node status, degree of malignancy, menopausal status, or age. Among complementary treatments, chemotherapies and hormone therapies are distinguished [121]. All these methods of treatment impose a heavy physical and psychological burden on the patient, are often associated with relapses, and carry complications in the form of oedema, malaise, nausea, or hair loss, which impair the patient’s quality of life. Therefore, medicine is constantly developing in search of new treatments that can target the cancer without damaging healthy tissues and the body as a whole. A chance for such a treatment may be offered by PDT, in that it is less invasive and more specific. The objective of this investigation was to assess the impacts of PDT with the disodium salt of PpIX on breast cancer tissue sections in vitro, employing MRI. In our study, we showed that PDT with PpIX disodium salt therapy significantly reduced the relaxation time on MRI examination of tumor cells in all 30 measurements, indicating that this therapy affects tumor cell death. The topic of PDT in breast cancer was also undertaken by Banerjee et al. [6]. In their research, the investigators employed PDT therapy with verteporfin on 26 patients to address a localized region of breast cancer slated for mastectomy, conducted a few days subsequent to PDT. In the mentioned study, the photosensitizer, verteporfin (0.4 mg/kg), was dissolved in a 5% dextrose solution and administered as a solitary intravenous infusion at a rate of 3 mL/min. Laser light with a wavelength of 690 nm and a power of 150 mW was utilized. The effects of PDT therapy were detected by MRI in seven patients. Among the six patients, it was possible to estimate the volume of PDT necrosis, which ranged from 78 to 8316 mm^3^, similar to our own study, where the application of PDT also had an effect on tumor cell necrosis. The authors’ conclusions are that PDT therapy under imaging guidance is a minimally invasive and promising treatment for primary breast cancer. Similar conclusions were also reached by Kong et al. [122]. The authors designed multifunctional ICG@SANPs-cRGD nanoparticles for the PDT of breast cancer. The authors conducted an in vivo study on mice and concluded that ICG@SANPs-cRGD-based PDT can inhibit cancer cell proliferation and migration and can also kill cancer cells through the induction of apoptosis and necrosis, similar to our own study where PDT therapy also had significant effects on cancer cell death, which was confirmed by MRI imaging and statistically significant results (*p* < 0.001). Other authors who have verified the effect of PDT on breast cancer include Ahn et al. [123]. The researchers used Photofrin (HpD) photosensitizer (5 mg/kg) and 350 J/cm^2^ to 30 J/cm^2^ laser light for PDT therapy. Breast cancer cells were maintained in culture medium and then injected into the then-prepared 24 mouse models. Tumor size ranged from 8 to 10 mm. According to the authors, the effects of the therapy depended on the laser energy used. Complete remission was confirmed 21 days after PDT with applied energy from 180 to 90 J/cm^2^, as in the 350 to 90 J/cm^2^ group. One in three mice showed no response in the group receiving 60 and 30 J/cm^2^. The authors concluded that the moderate energy needed to treat breast cancers <10 mm in size is about 90 J/cm^2^. The authors’ conclusions are consistent with our own, i.e., that with properly selected parameters, PDT affects tumor cell necrosis. The topic of PDT in killing breast cancer tumor cells was also investigated by Dos Santos et al. [124]. The researchers investigated the outcomes of PDT by utilizing methylene blue (MB-PDT) across three breast epithelial cell lines that represented benign conditions and various subtypes of breast tumors. The study revealed that the therapy elicited varying levels of extensive cell death in tumor cells, aligning with their own findings where PDT treatment also impacted tumor cell mortality. Notably, malignant cells exhibited higher susceptibility to the therapies in comparison to non-malignant cells. The authors conclude that PDT therapy has the potential to kill breast cancer tumor cells while remaining safe for healthy tissue. Another of the studies that tested the effect of PDT therapy on breast cancer in vitro was that of Aniogo et al. [125]. The study used a 681.5 nm diode laser with a power of 4.53 mW/cm^2^ for about 18 min. Cell viability was examined using a trypan blue test and a quantitative homogeneous adenosine triphosphate test. The authors showed that the combination treatment with doxorubicin and PDT was effective in inhibiting tumor cell growth and also increased the yield of apoptotic cells. The authors concluded that this form of therapy has potential in cancer treatment, although further research in this area is needed. The relaxation time of breast cancer cells is an important element in the diagnostics distinguishing healthy cells from cancer cells. In our work, it is possible to notice the differences between T1 and T2 in the tissue. Nissan N. et al. conducted a study with the objective of examining the clinical importance of lipid relaxation times in both typical fibroglandular tissue and breast carcinoma. They utilized magnetic resonance spectroscopic fingerprinting (MRSF) for imaging, involving a group of 12 patients diagnosed with breast cancer through biopsy and another group of 14 healthy individuals. The analysis revealed seven lipid metabolite peaks with significant differences in relaxation times between cancer and normal tissue at specific resonances (1.3 ppm, 4.1 ppm, and 5.22 ppm for T1, and 5.31 ppm for T2). The results suggest that MRSF is a feasible and time-efficient technique for assessing lipid relaxation times in breast cancer, potentially serving as quantitative markers for distinguishing normal and cancerous tissues [126]. Further research is needed to understand the biological mechanisms behind these differences.

## 5. Conclusions 

In all the cited studies, the authors reached similar conclusions, namely that PDT has the potential to cause cancer cell death while maintaining the safety of healthy tissue. Our own study also showed that the therapy significantly affected cancer cells, as confirmed by a significant reduction in the relaxation time of tumor cells on the MRI results, which offers potential for further research in this area. Our study, employing rigorous statistical analyses through Statistica software and graphical representation using Microsoft Excel and Word, brings novel insights to this domain. Utilizing MRI, we examined 30 samples comprising breast cancer tumor cells subjected to PDT. The detailed analysis of each sample, with 30 observations each, revealed distinct characteristics. Notably, the relaxation time of tumor cells, a crucial indicator assessed through T1 and T2 measurements, exhibited a statistically significant reduction post-therapy compared to pre-therapy measurements. This reduction was substantial, with a mean difference of 464.5 ms and a standard deviation difference of nearly 10 ms. These precise measurements, highlighted in Table 3 and Table 4 and visually represented in the box and whisker plot (Figure 3), provide robust evidence of the therapy’s impact on cancer cells. The data obtained in our research not only affirm the potential of PDT but also contribute unique and detailed insights that warrant further exploration in this critical area of cancer treatment. The novelty of this work lies in its meticulous statistical approach and the application of advanced imaging techniques to assess the impact of PDT on breast cancer tumor cells. Unlike previous research, which often employed basic statistical analyses, this study utilizes sophisticated tools, such as the Statistica software, Microsoft Excel, and Microsoft Word for in-depth statistical analysis and graphical representation of results. The use of the Shapiro–Wilk test to assess the normality of data spread is a methodological innovation, allowing for a more robust selection of appropriate statistical tests. The study examines 30 samples containing PDT-treated breast cancer tumor cells using MRI, providing a comprehensive dataset for analysis. Notably, the comparison of T1 and T2 measurements reveals statistically significant differences between pre- and post-therapy measurements, showcasing the effectiveness of PDT. The detailed presentation of descriptive statistics, including mean, standard deviation, median, and quartile values, adds a layer of precision to the findings. The emphasis on the difference in means and standard deviations, along with graphical representations, such as box and whisker plots, enhances the clarity and depth of the statistical analysis, setting this work apart in its rigor and methodological innovation compared to other publications in the field.

## Figures and Tables

**Table 1:** Molecular classification of breast cancer.

Molecular Subtype	ER (Estrogen Receptor)	PR (Progesterone Receptor)	HER2	Additional Markers
Basal-like [29]	−	−	−	CK5/6+, EGFR+
HER2/ER- [30]	−	−	+	CK5/6+/−,EGFR+/−
Luminal A [31]	+	+	−	Low levels of the protein Ki-67
Luminal B [32]	+	−	−	High levels of Ki-67
Normal breast-like [33]	−/+	Unknown	−	CK5/6+, EGFR+
Interferon-related [34]	−/+	Unknown	−	STAT1
Claudin-low [35]	−	−	−	CLDN low/−, CDH1 low/−, CK5/6 +/−, EGFR +/−
Molecular apocrine [36]	−	−	+/−	AR+, CK5/6+/−, EGFR+/−

**Table 2:** Benefits and drawbacks of different breast cancer treatment approaches.

Treatment Approaches	Benefits	Drawbacks
Surgery	- Can be curative for early-stage cancers. - Provides definitive pathology for accurate diagnosis. - May be combined with other treatments for better outcomes [79].	- May result in changes in breast appearance. - Possible complications, such as infection or lymphedema. - Limited effectiveness for advanced stages [80].
Radiation therapy	- Targets and kills cancer cells in a specific area. - Often used after surgery to reduce the risk of cancer recurrence. - Non-invasive and painless [81].	- Potential damage to nearby healthy tissues. - Skin irritation or changes in breast appearance. - Long treatment duration [82].
Chemotherapy	- Systemic treatment that reaches cancer cells throughout the body. - Effective against rapidly dividing cancer cells. - Adjuvant or neoadjuvant use for various cancer types [83].	- Side effects, such as nausea, fatigue, hair loss, and immunosuppression. - Impact on healthy rapidly dividing cells. - Limited efficacy against some types of breast cancer [84].
Hormone therapy	- Targets hormone receptor-positive breast cancers. - Reduces the risk of recurrence in hormone-sensitive tumors. - Often used for both early and advanced stages of cancer [85].	- Side effects including hot flashes, mood swings, and joint pain. - Resistance may develop over time. - Not effective for hormone receptor-negative tumors [86].
Targeted therapy	- Specifically targets cancer cells with minimal impact on normal cells. - Can enhance the effectiveness of chemotherapy. - Used for HER2-positive breast cancers [87].	- Resistance may develop. - Potential for side effects, such as heart problems. - Limited efficacy in HER2-negative tumors [88].
Immunotherapy	- Activates the immune system to target and destroy cancer cells. - Ongoing research for breast cancer treatment. - Potential for durable responses [89].	- Limited effectiveness in some breast cancer subtypes. - Immune-related side effects. - Currently not a standard treatment for all breast cancers [90].
Photodynamic therapy	- Minimally invasive. - Targeted treatment. - Limited side effects. - Can be repeated multiple times if necessary. - Cosmetic benefits [91].	- Limited tissue penetration. - Not suitable for metastatic cancer. - Sensitivity to light. - May not completely destroy all cancer cells. - Often used in combination with other treatments, such as surgery or chemotherapy, to achieve more comprehensive cancer control [92].

**Table 3:** Descriptive statistics for T1 and T2 measurements.

Variable	*N*	$\bar{x}$	*Me*	*Min.*	*Max.*	*Q1*	*Q3*	*SD*
Value T1 [ms]	30	545.27	545.5	505.0	598.0	516.0	570.0	29.69
Uncertainty T1 [ms]	30	43.83	48.0	4.0	80.0	20.0	72.0	25.83
Value T2 [ms]	30	80.77	83.5	34.0	117.0	69.0	95.0	19.80
Uncertainty T2 [ms]	30	16.33	15.5	1.0	55.0	8.0	21.0	10.63

**Table 4:** *t*-test result for variation in dependent measurements.

Variable	$\bar{x}$	*SD*	*N*	$\bar{x}$ *Difference*	*SD*	*t*	*df*	*p*
Value T1 [ms]	545.27	29.69	30	464.5	31.54	80.67	29	<0.001
Value T2 [ms]	80.77	19.80						

## Data Availability

All data have been included.
